# A new perspective for mitigation of SARS-CoV-2 infection: priming the innate immune system for viral attack

**DOI:** 10.1098/rsob.200138

**Published:** 2020-07-29

**Authors:** Oren Kolodny, Michael Berger, Marcus W. Feldman, Yoav Ram

**Affiliations:** 1 Department of Ecology, Evolution and Behavior, Alexander Silberman, Institute of Life Sciences, The Hebrew University of Jerusalem, 9190401 Jerusalem, Israel; 2 The Lautenberg Center for Immunology and Cancer Research, Institute of Medical Research Israel-Canada, The Hebrew University of Jerusalem–Hadassah Medical School, Israel; 3 Department of Biology, Stanford University, Stanford CA, USA; 4 School of Computer Science, Interdisciplinary Center Herzliya, Israel

**Keywords:** SARS-CoV-2, covid-19, priming, vaccines, innate immune system, trained immunity

## Abstract

The course of infection by SARS-CoV-2 frequently includes a long asymptomatic period, followed in some individuals by an immune dysregulation period that may lead to complications and immunopathology-induced death. This course of disease suggests that the virus often evades detection by the innate immune system. We suggest a novel therapeutic approach to mitigate the infection's severity, probability of complications and duration. We propose that priming an individual's innate immune system for viral attack shortly before it is expected to occur may allow pre-activation of the preferable trajectory of immune response, leading to early detection of the virus. Priming can be carried out, for example, by administering a standard vaccine or another reagent that elicits a broad anti-viral innate immune response. By the time that the expected SARS-CoV-2 infection occurs, activation cascades will have been put in motion and levels of immune factors needed to combat the infection will have been elevated. The infection would thus be cleared faster and with less complication than otherwise, alleviating adverse clinical outcomes at the individual level. Moreover, priming may also mitigate population-level risk by reducing need for hospitalizations and decreasing the infectious period of individuals, thus slowing the spread and reducing the impact of the epidemic. In view of the latter consideration, our proposal may have a significant epidemiological impact even if applied primarily to low-risk individuals, such as young adults, who often show mild symptoms or none, by shortening the period during which they unknowingly infect others. The proposed view is, at this time, an unproven hypothesis. Although supported by robust bio-medical reasoning and multiple lines of evidence, carefully designed clinical trials are necessary.

## 1. Introduction

Widespread vaccination capable of specifically neutralizing the SARS-CoV-2 virus is expected to provide the ultimate solution to the COVID-19 epidemic. However, a vaccine is still unavailable, and preventative medication is currently lacking [1,2]. We propose a novel therapeutic approach that to date has been under-explored in the COVID-19 epidemic: we suggest that priming an individual's immune system during active epidemic, by inducing a short-term anti-viral systemic activation of the innate immune system, may reduce the infection's severity, length and probability of complications.

Following viral infection, the innate immune system is activated when pattern-recognition receptors (PRRs) are engaged by microbe-associated molecular patterns (MAMPs) in viral proteins and nucleic acids [3,4]. Specifically, the endosomal toll-like receptors (TLRs) 3, 7 and 8, and the intracellular cytosolic PRRs, such as MDA5 and RIG-I, have been shown to respond to respiratory infection by RNA viruses such as coronaviruses [5–7]. These sensors recognize viral RNA, such as 5′ triphosphate single-strand RNA and double-stranded RNA, and trigger a downstream signalling cascade to ultimately induce the secretion of types I and III interferons (IFNs) and proinflammatory cytokines [4]. In turn, the IFNs stimulate their cognate receptors and induce the activation of thousands of interferon stimulated genes (ISGs) that establish an anti-viral state in the infected cells and in surrounding cells [8,9]. This state efficiently inhibits further spread of the infection, while allowing time for the activation of adaptive responses that in most cases will clear the virus from the infected individual. These cascading dynamics are also critical to guarantee sufficiently strong, but not excessive, innate and inflammatory immune responses, and a timely downregulation of these responses to protect the individual from harmful immunopathology [7].

Evidence so far suggests that in the course of COVID-19, the SARS-CoV-2 virus has an average incubation period of approximately 5 days, and up to 14 days and longer [10,11]. This long period, alongside recently published direct evidence [12], suggests that SARS-CoV-2 initially manages to evade the innate immune system during early stages of infection. Studies of related coronaviruses SARS-CoV and MERS-CoV have demonstrated that these viruses encode a large number of factors that delay or suppress anti-viral interferon responses and may be involved in the evasion of immune detection [5,12]. At later stages of the disease, uncontrolled viral replication triggers hyper-inflammatory conditions in some individuals, which can lead to induction of lung injury by a cytokine storm [12,13].

Therefore, we suggest that priming the anti-viral innate immune response prior to SARS-CoV-2 infection may trigger an enhanced anti-viral interferon response ahead of time, thus preventing immune evasion by the virus. This may direct the immune response towards the preferable route for overcoming COVID-19 and prevent the immune pathology seen in the more severe cases. We anticipate that the ensuing infection would be attenuated relative to the infection of a naïve unprimed individual, as has been shown in analogous murine model systems [14–18]. A primed infection would still allow the adaptive immune system to develop adaptive immunity to SARS-CoV-2. This adaptive immunity is required at a population scale to halt the epidemic.

Our proposal does not intend to prevent a primed individual from being infected, but—by readying the immune system ahead of time—to lessen the severity of the infection and risk of complications, and to shorten the duration of infection. At the population level, the shortened duration of infection could change the epidemic dynamics, helping to ‘flatten’ the epidemic curve and to reduce the maximal number of infected and hospitalized individuals at any time point [19,20]. To alter the population-level dynamics in this way, the infection-shortening aspect of our proposal may be important even in subclinical and asymptomatic individuals, as they are likely to be infectious and play a major role in the spread of the epidemic [21].

The ‘gold standard’ of the immune response to a pathogen is often perceived solely as the presence (or absence) of specific antibodies and T cells that allow the adaptive immune system to identify and neutralize the pathogens, and, for viruses, to kill infected cells. This traditional focus creates a misleading impression regarding the immune system's ‘bread and butter’ function: alongside components of the adaptive immune system that provide a response which is specific to a particular pathogen, there are thousands of genes that are involved in anti-viral defence and that are not pathogen-specific [22–25]. This is reflected in the extensive overlap between the sets of proteins whose production is upregulated in response to different viral infections [24,26–32].

Defence priming—upregulation of immune function in response to environmental cues, social cues or physiological cues emitted by conspecifics—is well known in plants and invertebrates [33–40]. For example, termites increase their production of immune-related proteins following interaction with nest mates that had been exposed to a pathogen [40]. Defence priming has also been shown in vertebrates, in particular via activation of components of the innate immune system [35,37,41–49]. Specifically, it has been demonstrated experimentally that activation of the mammalian immune system by various triggers, from social cues to exposure to microbes or microbially derived compounds, provides protection upon exposure to an unrelated pathogen [14–18,50–57]. For example, mice that were administered aerosolized bacterial lysate exhibited an innate immune response—increased cytokine levels—and survived an otherwise lethal exposure to Influenza A [17]. Priming of the immune system by exposure to agents other than the pathogen itself is common: priming and upregulation of the immune system by the mammal's commensal bacteria have been frequently suggested and its importance has been repeatedly demonstrated [58–62].

Most encouraging are recent experiments on priming the immune system for intermediate time scales in humans: Arts *et al*. [63] have recently shown that BCG vaccination against tuberculosis activates factors of the innate immune system for extended periods of time, on the order of weeks, and increases resistance to an experimental infection by an attenuated yellow fever virus. This phenomenon, dubbed ‘trained immunity’ or ‘innate immune memory’ [44,64–66], relates to the longer-lasting effects of priming the immune system, but supports the feasibility of the short-term priming that we propose here. Similarly, a decreased rate of non-targeted infections has been reported in children in the period following vaccination for measles, mumps and rubella, as well as following vaccination with live-attenuated polio virus [67–70]. A number of studies are currently exploring the potential attenuation or prevention of COVID-19 via vaccination with BCG or MMR vaccines [71–74].

Different triggers may serve to prime the immune system, readying it for attack by stimulating it to mount a short-term broad anti-viral response. Priming with bacteria and bacterially derived factors, particularly administered nasally, has been shown experimentally to significantly alleviate the severity of viral challenges that attack the respiratory system [14–18,50–52,56,57]. An even more promising category of priming agents are attenuated viruses used in vaccines, various virus-derived elements, virus-like particles and other components [63,64,71,75–80]. Such agents have been studied and tested extensively, and candidates have been highlighted specifically for their ability to trigger a broad anti-viral immune response, acting as adjuvants in anti-viral vaccines. The systemic priming can be carried out using various therapeutic agents, including many off-the-shelf products and common vaccines that are prescribed prophylactically such as influenza, polio or varicella-zoster vaccines [81–85]. Use of vaccines as triggers in such a context would aim to capitalize on the broad innate immune response that they trigger and which is transient, lasting for a number of days to a few weeks and possibly longer after administration [63,81–86]. The longer-lasting effect of gaining adaptive immunity to the specific virus or viral strain that the vaccine is designed for would be a potentially beneficial unrelated side effect and would not be expected to play a role in countering SARS-CoV-2.

Decreased incidence and rate of complications of COVID-19 have been reported in children. In the light of our proposal, this might be partially attributed to immune system priming, caused by the frequent rate of vaccination during childhood in many countries. Similarly, negative correlations have been reported between COVID-19 prevalence and severity of outcomes, and region-level prevalence of malaria, helminths and schistosomiasis [87–90]. These infectious agents, whose prevalence correlates positively with the prevalence of other infectious diseases, may have an immunomodulatory effect which primes the immune system for viral attack. This possibility warrants careful exploration. Finally, it has recently been suggested that there is a negative correlation between coverage of influenza vaccination and deaths from COVID-19 in the elderly [91], presenting another promising observation that may support our proposed perspective.

**Figure 1.**
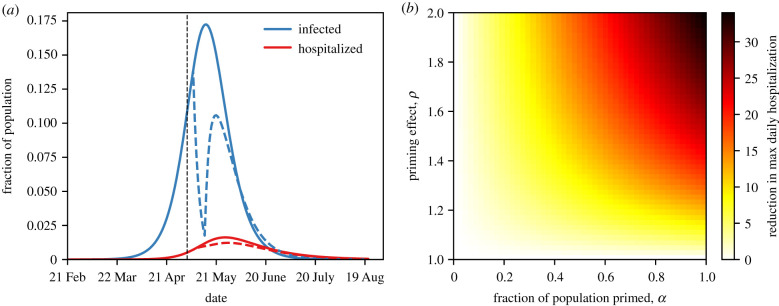
Effect of priming on epidemic dynamics. (*a*) Fraction of infected (blue) and hospitalized (red) individuals in the population over time without priming (solid lines) or with priming (dashed) if priming were administered on 5 May (day 72) to the entire population (fraction of primed individuals *α* = 1), assuming the effect of priming *ρ* lasts for one week and that it reduces the infectious period and chance of hospitalization by *ρ* = 1.5 (i.e. by 33%). (*b*) Reduction in maximum daily hospitalizations due to priming for various fractions of priming *α* (on the x-axis) and effects of priming *ρ* (on the y-axis). Dynamics are based on an SEIR model where infected individuals are primed with probability *α* and otherwise not primed. Model parameters estimated by Pei & Shaman from US county-level incidence data between 21 February and 13 March 2020 ([92], table 3): transmission rate *β* = 0.635 (weighted average of documented and undocumented cases); expected latency period *δ^−1^* = 3.59 days; expected infectious period *r^−1^* = 3.56 days, or *rρ* if primed in the past week. An additional model compartment for hospitalized individuals was added: infected individuals are hospitalized at rate *h* = 0.014 per day [93], or *h/ρ* if primed in the past week, for an expected duration of *γ^−1^* = 21 days [93]. See https://github.com/yoavram-lab/ImmunePriming for Python source code.

To demonstrate the potential population-level impact of our proposal, we have incorporated large-scale population priming in an SEIR model that has been used to analyse and forecast the COVID-19 epidemic trajectory in China and the continental US [21,92], using parameters previously estimated from US county-level data between 21 February 2020 and 13 March 2020 [92]. Figure 1*a* shows the fraction of infected and hospitalized individuals with and without priming. If priming reduces the infectious period and chance of complications by 33%, the priming agent is administered to the whole population slightly before infection rates peak, and priming is effective for a week, the maximum number of hospitalized individuals is reduced by 25%. Figure 1*b* explores such reductions in hospitalizations for different parameter combinations: the fraction of the population receiving the priming agent, and the factor by which priming reduces the infectious period and chance of complications that require hospitalization. Although this is a simplified model (e.g. only a single population is examined rather than a metapopulation as in [21,92]), it demonstrates the potential population-level effect that priming might have on the epidemic trajectory and its impact in a city, region or state.

A number of caveats are associated with our proposal. First, it is crucial that the priming does not evoke an autoimmune response. In this respect, authorized therapeutic agents such as broadly used vaccines are preferable as a first set of candidates. Second, it is necessary to test and choose priming agents that do not trigger an adverse effect (i.e. to ensure that they do not burden the immune system and make it less effective in countering the ensuing attack by SARS-CoV-2). Finally, many of the cases of severe symptoms and mortality of COVID-19, especially in the elderly, seem to involve a cytokine storm of hyper-inflammation, in which much of the damage is caused by the immune system itself [13,94,95]. It is important to ascertain whether the proposed activation of the immune system prior to infection would reduce the likelihood of immune system dysregulation and hyper-inflammation. Evidence from murine models is encouraging: the viral challenges used were characterized by a tendency to stimulate a hyper-inflammatory condition often accountable for the major damage to the host; the primed individuals in these experiments suffered significantly less from such complications than the control groups [14,15,17,52,56,57]. A particular risk–benefit exploration needs to be carried out for the elderly, who are at the greatest risk for severe and lethal complications of COVID-19 [96–98]. The reduced efficacy of immune functions involved with aging [99–101] raises the concern that priming would burden their system further and reduce its ability to respond to the SAR-CoV-2 infection. However, for the same reason, early preparation of the immune system to the expected attack may be crucial and beneficial for the elderly. This may be specifically true with respect to early activation of toll-like receptors such as TLR7 that may detect SARS-COV-2 infection and trigger an appropriate immune response [5–7]. Carefully designed clinical trials will be necessary to determine the risks and opportunities of the approach we propose for the elderly.

The COVID-19 epidemic is a rare case of a rapidly spreading epidemic that can reach high infection levels in affected populations. Although this poses a major challenge, it also constitutes an Achilles' heel that can be used to attenuate the epidemic's devastating effects: once the virus has spread in a population (e.g. a specific town or city), the timing of infection for many individuals is highly predictable. Similarly, large-scale infection may be expected shortly following removal of a population lockdown, or specifically among individuals that return to the work-force when a general lockdown is gradually lifted. Our approach capitalizes on the predictability of the infection and suggests a way to prepare susceptible individuals to counter the expected attack. Even vaccines that are often prescribed without particular medical indication, such as MMR, the polio vaccine or the seasonal flu vaccine, might serve this purpose.

In light of the imminent threat posed by SARS-CoV-2 to millions around the world and the current lack of preventative therapeutic measures, our proposal could be highly beneficial. It can potentially be implemented using extant authorized therapeutic agents such as broadly used vaccines for viral diseases, and thus may involve relatively low risk and can be readily tested. Our proposal combines direct individual-level effects—reducing complication rates, hospitalization events and mortality—and effects that play out at the population level—reduction of the infectious period, including of asymptomatic yet infectious individuals, and reduction of peak hospitalization load. Given the scale of the challenge that humanity is facing, even a moderate attenuation of the duration, severity, and complication risk of COVID-19 infections may, via these direct and indirect effects, would save many lives.

## Data accessibility

This article has no additional data.
